# The Role of Extracellular Vesicles in Mediating Resistance to Anticancer Therapies

**DOI:** 10.3390/ijms22084166

**Published:** 2021-04-17

**Authors:** Saeideh Maleki, James Jabalee, Cathie Garnis

**Affiliations:** 1 Postgraduate Program in Interdisciplinary Oncology, Department of Integrative Oncology, British Columbia Cancer Research Centre, Vancouver, BC V5Z 1L3, Canada; smaleki@bccrc.ca (S.M.); jjabalee@bccrc.ca (J.J.); 2 Department of Surgery, University of British Columbia, Vancouver, BC V5Z 1M9, Canada

**Keywords:** extracellular vesicles, cancer, chemoresistance, tumor microenvironment, miRNA

## Abstract

Although advances in targeted therapies have driven great progress in cancer treatment and outcomes, drug resistance remains a major obstacle to improving patient survival. Several mechanisms are involved in developing resistance to both conventional chemotherapy and molecularly targeted therapies, including drug efflux, secondary mutations, compensatory genetic alterations occurring upstream or downstream of a drug target, oncogenic bypass, drug activation and inactivation, and DNA damage repair. Extracellular vesicles (EVs) are membrane-bound lipid bilayer vesicles that are involved in cell–cell communication and regulating biological processes. EVs derived from cancer cells play critical roles in tumor progression, metastasis, and drug resistance by delivering protein and genetic material to cells of the tumor microenvironment. Understanding the biochemical and genetic mechanisms underlying drug resistance will aid in the development of new therapeutic strategies. Herein, we review the role of EVs as mediators of drug resistance in the context of cancer.

## 1. Introduction

**Figure 1:**
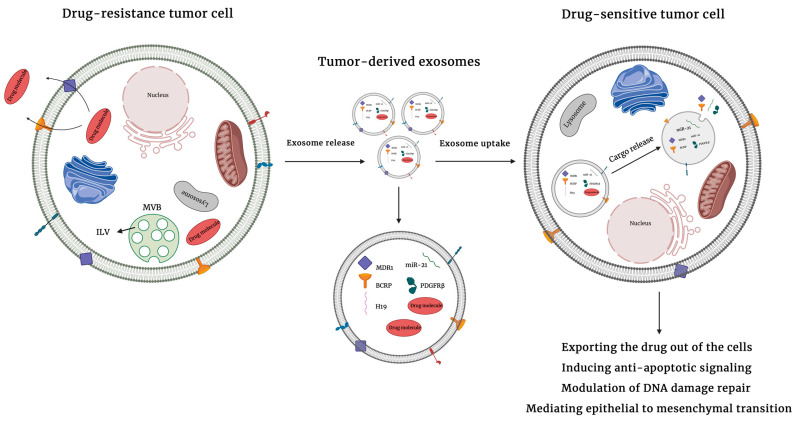
Mechanisms of extracellular vesicles-mediated drug resistance. Extracellular vesicles can induce drug resistance by directly transferring bioactive cargo molecules including drug efflux pumps, inhibitors of apoptosis, and prosurvival molecules to neighboring cells as well as cells in the tumor microenviroments. The uptake of cargo by recipient cells promotes drug resistance by exporting drugs out of cancer cells, inducing anti-apoptotic signals, modulating DNA damage repair, or mediating epithelial to mesenchymal transition.

Cancer is a major global public health issue and is the second leading cause of death worldwide. It is estimated that by the end of 2020, 1,806,590 new cases and 606,520 deaths will occur in the United States due to cancer [1]. Chemotherapy is a prominent treatment modality for many cancer patients. Chemotherapeutic drugs are cytotoxic agents whose mechanism of action is not limited to cancer cells and thus result in a plethora of negative side-effects. Commonly, chemotherapeutics work by interfering with DNA synthesis and DNA repair pathways. Improved knowledge of tumor complexity has driven the development of therapies targeted to specific genes and mutations. Targeted therapy inhibits cancer growth by interfering with specific molecules that are required for tumorigenesis, thus allowing treatment to be personalized according to the genetics of a given tumor [2]. However, the development of resistance to therapeutic options—including both chemotherapy and targeted therapies—constitutes a fundamental challenge in cancer treatment which leads to treatment failure in over 90% of patients with metastatis disease [3]. Drug resistance involves a reduction in potency and efficacy of a drug and can lead to failure in achieving therapeutic goals, which in turn drives decreased survival. It can exist before the use of a therapeutic agent (intrinsic resistance) or can arise during treatment (acquired resistance) [4]. Mechanisms underlying drug resistance are complex, and include drug efflux as an intrinsic resistance mechanism, secondary mutations that alter drug binding or kinetics, compensatory genetic alterations influencing downstream targets or upstream effectors of a specific gene or protein, oncogenic bypass, drug activation and inactivation, and DNA damage repair as acquired resistance mechanisms [5]. Recent research suggests a key role for extracellular vesicles (EVs), small membrane-bound vesicles that transfer cargo molecules amongst cells of the tumor and tumor microenvironment, in regulating the development of drug resistance (Figure 1).

## 2. Role of Extracellular Vesicles in Drug Resistance

### 2.1. Extracellular Vesicles

Cell–cell communication is a key feature of cancer progression and metastasis. Interactions among tumor cells, cells of the tumor microenvironment, and cells at distant locations is required to provide a hospitable pre-metastatic niche and to promote migration, invasion, and drug resistance in disseminated tumor cells [6,7]. Much attention has been given to the role of EVs in mediating this cross-talk [6]. EVs are a heterogenous group of lipid bilayer structures derived from either endosomal multivesicular bodies (exosomes) or the plasma membrane (microvesicles, also called ectosomes) [8]. They regulate intercellular communication by transferring various cell-type specific biomolecules (including proteins, lipids, and nucleic acids) amongst cells [8]. Uptake of such biomolecules into the recipient cell results in a change in phenotype that may promote tumorigenesis [9,10].

### 2.2. Extracellular Vesicle Biogenesis

**Figure 2:**
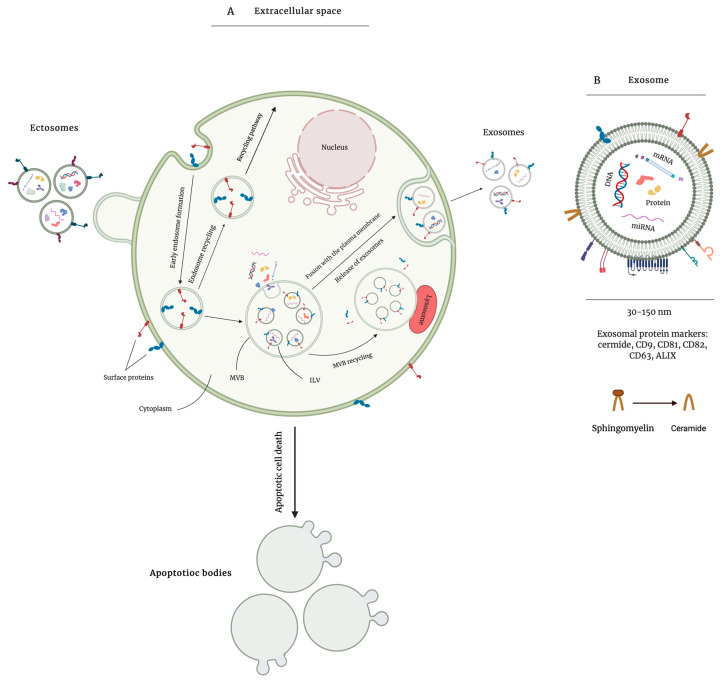
Overview of extracellular vesicle (EV) biogenesis. EVs are lipid bilayer vesicles that are secreted by almost all cell types and are associated with mediating biological processes. There are three types of EVs based on their biogenesis, morphology, size, and isolation method. EVs are most commonly isolated via differential ultracentrifugation at different speeds. Apoptotic bodies, as the largest group of EVs, are secreted by cells undergoing apoptosis and are pelleted at ~2000× *g*. Ectosomes are formed through outward blebbing of the plasma membrane and are pelleted at ~10,000× *g* (**A**). Exosomes are formed through invagination of the multivesicular bodies with the plasma membrane and contain several biomolecules such as proteins, DNA, and different types of RNA. They include CD9, CD81, CD63, and ALG-2-interacting protein X (ALIX) as surface protein markers and are pelleted at high speed ultracentrifugation (**B**).

Based on the site of biogenesis, morphology, and size, EVs can be categorized into three main populations: exosomes, microvesicles (MVs), and apoptotic bodies. Exosomes are vesicles of a relatively homogeneous size, ranging from 30 to 150 nm in diameter, and are formed through endosomal trafficking—a process in which exosomes form as intraluminal vesicles (ILVs) within multivesicular bodies (MVBs) [8] (Figure 2). Upon the formation of exosomes, Rab-guanosine triphosphatases (Rab-GTPases) are recruited to regulate the fusion of MVBs with the plasma membrane and release exosomes [11,12]. Exosome biogenesis depends on the endosomal sorting complex required for transport (ESCRT) machinery, which is responsible for ILV formation and protein sorting. ESCRT is made up of more than 30 proteins that are assembled into four protein complexes (ESCRT-0, -I, -II, and -III) and associated proteins vacuolar protein sorting-associated protein 4 (VPS4) and ALG-2-interacting protein X (ALIX). ESCRT-0, ESCRT-I, and ESCRT-II are responsible for recognizing and sequestering ubiquitin-modified endosomal membrane proteins. ESCRT-III generates inward budding structures by completing the scission of ILVs and ALIX facilitates cargo clustering from the plasma membrane [13]. MVBs and exosomes can also form independently of ESCRT machinery, in a ceramide-dependent or tetraspanin-dependent manner [14,15]. There are several methods that are used to isolate EVs. However, the most common method is differential ultracentrifugation in which contaminating material are removed through centrifugation at low speeds and EVs are pelleted at higher speeds (~100,000× *g*) [16]. Exosomes are formed via invagination of the multivesicular body, range in size from 50 to 200 nm, and are pelleted at ~110,000× *g*. Ectosomes (also known as microvesicles), that are formed through outward blebbing of the plasma membrane, range in size from 50 to 1000 nm and are pelleted at ~10,000× *g* [17]. Apoptotic bodies are 500 to 5000 nm vesicles released by cells undergoing apoptosis and pelleted at ~2000× *g* [6]. Herein, we mostly refer to works done on EVs extracted through differential ultracentrifugation (Table 1).

### 2.3. Selection of Exosome Content

While some EV cargo is passively packaged into the EVs, there is some evidence for selective packaging as well [18]. Proteins are major components of EVs which not only influence the phenotype of the recipient cells, but also determine the destination of EVs [18]. Specific EV protein markers bind to and activate receptors on certain recipient cells and release the EV content into the cells. The sorting of proteins into exosomes at least partly depends on protein ubiquitylation and the ESCRT machinery [18]. Ubiquinated proteins are recognized by ESCRT–0 which recruits ESCRT–I and ESCRT–II and this complex of ESCRT–0, -I, and -II recruits ESCRT–III and initiates protein sorting into ILVs [6]. Apart from ESCRT-dependent pathways, phosphorylation is another mechanism of sorting proteins into EVs, with studies showing evidence of the role of EPHA2 and AGO2 in promoting or inhibiting protein sorting, respectively [19,20]. Additionally, recruiting certain proteins via other proteins such as tetraspanins (including CD9, CD63, CD81, and CD82) as well as dimerization are considered to be involved in sorting proteins into EV [21,22].

In addition to proteins, exosomes carry nucleic acids including DNA, mRNA, circular RNAs (circRNAs), and non-coding RNA (ncRNA) [23]. ncRNAs are RNA molecules that exhibit no protein-coding potential. Two major types of ncRNAs that are packaged into exosomes include microRNAs (miRNAs) and long non-coding RNAs (lncRNAs) [24]. miRNAs are small ncRNAs containing 20–22 nucleotides that comprise the major RNA content of exosomes. miRNAs bind to the 3′-untranslated region (3′-UTR) of target mRNAs and regulate their expression by either degrading or inhibiting their transcription, thus affecting cell functions and modulating cell signaling pathways [25]. lncRNAs are ncRNAs containing > 200 nucleotides and are the second major RNA component of exosomes. lncRNAs are involved in the regulation of gene expression [26]. Although the mechanism of sorting RNA into exosomes is not fully understood, there are some factors that have been shown to play a role. RNA binding proteins (RBP) are responsible for the recognition of certain miRNA motifs and selectively sorting miRNAs into EVs [27]. For instance, the RBP SYNCRIP has a high affinity for miRNAs containing GGCU in the 3′ region of their sequence, whereas the RBP hnRNPA2B1 was found to bind to GGAG motif-containing miRNAs [27,28]. The ribonucleoprotein–miRNA (RNP–miRNA) complex is then sorted into EVs [29]. The RBP-mediated sorting of exosomal miRNAs can affect tumorigenesis based on the oncogenic or tumor suppressive function of the miRNA [30]. Another factor regulating miRNA sorting is 3′-end nucleotide additions (NTAs). miRNAs that have 3′-end uridylation have poor activity and decreased levels of interaction with their mRNA targets and thus tend to be specifically sorted into EVs [31]. However, since adenylation stabilizes miRNAs, those with adenylated 3′-end are mainly represented in cells allowed to interact with their targets [32,33]. Ceramide has also been show to play a role in miRNA sorting. Ceramide is a membrane lipid that is the product of the breakdown of sphingomyelin via neutral sphingomyelinases (nSMases) [34]. Inhibiting the generation of ceramide using nSMase inhibitor GW4869 not only decreases the biogenesis and release of EVs, but also reduces the amount of small RNA in EVs [35]. Ceramide forms ceramide-rich microdomains and facilitates the interaction of these domains with miRNAs in the MVB membrane [36]. MiRNAs with certain sequences and a higher affinity for ceramide are selectively packaged into EVs [36]. External factors such as carcinogens, oncogenic viruses, and hypoxia can also influence the miRNA content of EVs [37,38].

### 2.4. Role of EV Protein Cargo in Mediating Drug Resistance

#### 2.4.1. Drug Efflux

Drug efflux is the main intrinsic resistance mechanism that allows cells to resist the effects of anticancer agents by removing them from the cell. Various cell membrane transporter proteins play a role in chemoresistance by promoting drug efflux. For instance, members of the ATP-binding cassette (ABC) protein superfamily regulate the flux of multiple chemotherapeutic agents across the plasma membrane. There are three main members of the ABC protein superfamily that regulate drug efflux, including ABCB1 (also known as P-glycoprotein and MDR1), ABCC1 (also known as MDR-associated protein 1—MRP1), and ABCG2 (also known as breast cancer resistance protein—BCRP). These molecules prevent the accumulation of chemotherapeutic drugs by eliminating these hydrophobic molecules from tumor cells [43]. ABCB1 is a large, substrate-specific, membrane-bound glycoprotein responsible for transporting a variety of cytotoxic drugs [44]. Elevated levels of ABCB1 result in reduced intracellular concentrations of chemotherapeutic drugs by pumping lipophilic agents out of cells, thereby inducing drug resistance [45].

Some tumor cells are known to package ABCB1 in their secreted EVs, thus allowing it to be delivered to nearby and distant cells where it modulates drug resistance [46]. Exosomes derived from doxorubicin-resistant breast cancer cell lines induce drug resistance to sensitive cells and this occurs by exosomal transfer of ABCB1 from resistant cells to sensitive ones. Uptake of exosomal ABCB1 by doxorubicin-sensitive cells allows doxorubicin to be pumped out of the cells more efficiently and greatly increases doxorubicin resistance [46]. This mechanism is further modified by additional factors. For instance, the transient receptor potential channel 5 (TrpC5) is responsible for increased levels of EV formation and secretion in doxorubicin-resistant breast cancer cells [47]. Intercellular transfer of TrpC5 to recipient cells via circulating EVs stimulates the production of ABCB1 and thus increases drug resistance in doxorubicin-sensitive cells [47]. Further, in nude mice bearing doxorubicin-resistant tumor xenografts, there is increased expression of TrpC5 proteins and high levels of TrpC5-positive circulating EVs, as compared to doxorubicin-sensitive tumor xenografts. The role of TrpC5 is also clinically relevant; for example, in breast cancer patients undergoing treatment with doxorubicin, TrpC5 expression levels are significantly higher in those with progressive disease compared to patients with partial or complete response [47].

The protein ubiquitin carboxy-terminal hydrolase L1 (UCH-L1) has also been found to play a role in regulating multidrug resistance. UCH-L1 enhances multidrug resistance and upregulates ABCB1 expression via activation of the mitogen-activated protein kinase/extracellular receptor kinase MAPK/ERK signaling pathway in human breast cancer cells [48]. Extracellular vesicles isolated from doxorubicin-resistant breast cancer cells carry high levels of UCH-L1 and ABCB1 [48]. Co-culturing these exosomes with doxorubicin-sensitive cells transmits the chemoresistance phenotype to recipient cells in a time-dependent manner. More importantly, blood samples collected from breast cancer patients exhibit a significant negative correlation between the amount of circulating UCH-L1-containing exosomes and the clinical outcome of chemotherapy [48]. In conclusion, several proteins have been identified that are transferred from drug-resistant to drug-sensitive cells via EVs and result in an increase in drug resistance in recipient cells via an increase in drug efflux.

#### 2.4.2. Compensatory Genetic Alterations

Genetic mutations can induce development of drug resistance through dysregulation of proteins upstream or downstream of a therapeutic target. Transmission of specific cargo to the tumor microenvironment (TME) via EVs can result in the activation of signaling pathways and the development of drug resistance in cancer cells.

Mutations in the BRAF (v-Raf murine sarcoma viral oncogene homolog B) oncogene are observed in ~50% of metastatic melanomas, with over 90% of those mutations resulting in a glutamic acid–valine substitution at codon 600 (BRAF V600E) [49,50]. Activating BRAF mutations drives tumorigenesis through the oncogenic MAP kinase/ERK pathway [51]. In BRAF mutant melanoma cells, exosomal transfer of proteins to the neighboring cells activates the PI3K/AKT signaling pathway and induces resistance to BRAF kinase inhibitors in patients harboring the BRAF V600E activating alteration. One such protein is platelet-derived growth factor receptor beta (PDGFRβ) [52]. This protein can be transferred to BRAF inhibitor responsive cells via exosomes, where it activates the oncogenic signaling pathway, PI3K/AKT, in a dose-dependent manner and rescues recipient cells from MAPK pathway BRAF inhibition [52]. Similarly, a novel truncated but functional form of the ALK (anaplastic lymphoma kinase) protein (ALK^RES^) can be incorporated into exosomes secreted from melanoma cells and transported to cells of the tumor microenvironment. ALK^RES^ disseminates resistance to the BRAF inhibitor vemurafenib in cancer cells by activating the MAPK signaling pathway [53]. Knockdown of ALK induces apoptosis and restores BRAF inhibitor sensitivity in previously resistant cells. Combined treatment with vemurafenib and ALK inhibitors efficiently decreases tumor burden in mice bearing ALK-positive melanoma tumors [53]. These results demonstrate the importance of EV-mediated protein transfer in mediating drug resistance in cells of the TME.

In addition to altering signaling pathways, transmission of proto-oncogenes via exosomes contributes to the development of drug resistance by promoting migration and invasion in cancer cells. For instance, exosomes released by glioblastoma multiforme (GBM) cells harboring the PTPRZ1-MET fusion (ZM fusion) have high levels of the proto-oncogene MET and induce resistance to the alkylating agent temozolomide by promoting migration, invasion, and epithelial-mesenchymal transition (EMT) in GBM cells [54]. The uptake of exosomes isolated from ZM fusion-bearing cells leads to increased MET and phosphorylated MET levels in recipient cells, thus promoting drug resistance. Glioblastoma patients harboring ZM fusion tumors are resistant to temozolomide therapy, while those harboring non-ZM fusion tumors receiving chemotherapy have prolonged overall survival and respond well to temozolomide [54]. Thus, genetic mutations in cancer cells can alter EV cargo in such a way that increases migration, invasion, and drug resistance in cancer and TME cells.

### 2.5. Role of Nucleic Acid Cargo in Mediating Drug Resistance

#### 2.5.1. Compensatory Genetic Alterations

##### Long Non-Coding RNAs

The presence of various types of RNA cargo in exosomes derived from both normal and cancer cells has been confirmed via next-generation sequencing studies [55]. Exosomal transfer of RNA molecules, including lncRNAs, miRNAs, and circRNAs, induces drug resistance in recipient cells by transmitting active biomolecules to neighboring cells in order to regulate certain genes and their corresponding signaling pathways [56]. As noted, genetic abnormalities cause cells to develop drug resistance by dysregulating proteins upstream or downstream of a drug target. lncRNAs, which are > 200 bp single-stranded RNAs that function in the regulation of gene expression, have been found to play a similar role. For instance, lncRNA H19 plays a role in chemoresistance to several chemotherapeutics, including doxorubicin, gefitinib, and erlotinib [57]. H19 is highly expressed in exosomes derived from breast and non-small cell lung cancer (NSCLC) cells, and depletion of H19 with small interfering RNAs (siRNAs) restored doxorubicin and gefitinib sensitivity to drug-resistant cells of each of these cancer types, respectively [57,58]. Exosomal transmission of H19 from parental cells that are resistant to gefitinib promotes gefitinib resistance in recipient cells [57]. H19 has also been shown to be highly expressed in erlotinib-resistant NSCLC cells and their secreted exosomes. The uptake of these exosomes by recipient cells results in H19-mediated downregulation of miR-615-3p and upregulating autophagy-related protein 7 (ATG7) expression [57]. ATG7 is an essential regulator of autophagy, a process involved in tumor suppression, maintenance of the stemness properties of cancer cells, disease recurrence, and anticancer drug resistance, and ATG7 upregulation in recipient cells induces resistance to erlotinib [57,59].

Studies suggest that exosomal transmission of H19 may be a major regulator of chemoresistance in several cancer types. In addition to cancer-derived EVs, lncRNA H19 is also found in EVs of tumor stromal cells. In colorectal cancer (CRC), H19 is upregulated and enriched in exosomes derived from cancer-associated fibroblasts (CAFs). Here, H19 was found to act as a competing endogenous RNA (CeRNA) sponge for miR-141, a known inhibitor of cancer cell stemness, and to activate Wnt/β-catenin signaling in recipient cancer cells [60]. In addition, CAF-derived exosomal H19 was found to promote oxaliplatin resistance in recipient cancer cells in vitro and in vivo [60].

While H19 is perhaps the best studied example, several other lncRNA species have also been found to promote resistance to various chemotherapeutics. For example, HOXA transcript at the distal tip (HOTTIP) and HNF1A1/AS1 have been found to increase resistance to the alkylating agent cisplatin in gastric and ovarian cancers, respectively [61,62]. Interestingly, these lncRNAs function by acting on different targets. HOTTIP functions as a sponge for miR-218, which has been found to have tumor suppressive functions in gastric cancer (GC) [63,64,65]. The inhibition of miR-218 in gastric cancer cells resulted in the activation of the oncoprotein HMGA1 and an increase in drug resistance [61]. Interestingly, additional work on the role of miR-218 in gastric cancer found that it was also capable of activating Hedgehog signaling via targeting of Smoothened, resulting in increased sensitivity to another alkylating agent, oxaliplatin [66]. In conrast to HOTTIP, HNF1A1/AS1 was found to sponge another tumor suppressive microRNA, miR-34b. This microRNA, which is commonly silenced by methylation in gastric cancer cells, is a direct target of p53 signaling and its activation results in apoptosis, senescence, cell cycle arrest, and a decrease in proliferation, migration, and metastasis [67,68,69]. Inhibition of miR-34b in ovarian cancer cells correlates with an increase in oncogenic tuftelin1 (TUFT1), a promoter of malignant progression in ovarian cancer [62,70]. Importantly, both HOTTIP and HNF1A1/AS1 have been found in the EVs of drug-resistant cancer cells [62,70]. Transfer of these lncRNAs to recipient cells has been reported to promote chemoresistance, and knockdown of either HOTTIP or HNF1A1/AS1 has been reported to promote cisplatin sensitivity [61,62]. Significantly, serum levels of exosomal HOTTIP have been reported to be significantly upregulated in patients exhibiting resistance to chemotherapy [61].

Additional lncRNAs such as MSTRG.292666.16, RP11-838N2.4, and Prostate Androgen-Regulated Transcript 1 (PART1) have been found to increase resistance to the epidermal growth factor receptor (EGFR) tyrosine kinase inhibitors (TKIs) osimertinib, erlotinib, and gefitinib, , respectively. In NSCLC, EVs expelled from osimertinib-resistant cells have significantly higher expression of lncRNA MSTRG.292666.16 in comparison with osimertinib-sensitive cells. Treatment of cells with osimertinib resulted in an upregulation of miR-21, miR-125b, TGF-β, and ARF6, and downregulation of the protooncogene c-Kit; however, co-incubation of cells with EVs derived from osimertinib-resistant cells inhibited these gene expression changes [71]. Additionally, knockdown of MSTRG.292666.16 restores sensitivity to osimertinib-resistant cells [71]. A similar phenomenon has been observed for erlotinib-resistant NSCLC cell lines that overexpress lncRNA RP11-838N2.4 [72]. Incorporation of RP11-838N2.4 into exosomes facilitates transfer to parental cells and thus disseminates drug resistance in the tumor microenvironment, and silencing of RP11-838N2.4 via Forkhead box protein O1 (FOXO1) restores drug sensitivity to resistant cells [72]. Moreover, gefitinib-resistant esophageal squamous cell carcinoma (ESCC) cells express high levels of lncRNA Prostate Androgen-Regulated Transcript 1 (PART1). Signal transducer and activator of transcription 1 (STAT1), a key mediator of cell death, activates PART1 by binding to its promoter region and driving transcription [73]. Overexpression of STAT1 significantly increases PART1 expression levels and inhibits gefitinib-induced cell death, while STAT1 knockdown decreases PART1 levels in gefitinib-resistant cells [73]. Elevated PART1 functions as a CeRNA and binds competitively to miR-129, which has been found to act as a tumor suppressor miR in ESCC, resulting in increased expression of the anti-apoptotic protein Bcl-2 [73]. Incorporation of extracellular PART1 into EVs increases gefitinib resistance in sensitive cancer cells both in vitro and in nude mouse models [73]. Both RP11-838N2.4 and PART1 are elevated in the serum of patients that respond poorly to treatment, suggesting potential roles as biomarkers or drug targets [72,73].

Finally, a trio of lncRNAs have been found to regulate trastuzumab resistance in Human Epidermal Growth Factor Receptor positive (HER2+) breast cancer cells, including Actin Filament Associated Protein 1 Antisense RNA1 (AFAP1-AS1), AGAP2-AS1, and small nucleolar RNA host gene 14 (SNHG14). Each of these lncRNAs is highly expressed in trastuzumab-resistant cells in comparison with corresponding parental cells, and uptake of lncRNA-containing EVs by recipient cells increases drug resistance whereas knockdown decreases resistance [74,75,76]. AFAP1-AS1 functions by binding with AU binding factor 1 (AUF1) and enhancing HER2 gene translation, whereas SNHG14 functions by activating expression of the anti-apoptotic Bcl-2 pathway [74]. Although a mechanism was not described for AGAP2-AS1, it was found that its sorting into EVs was regulated by heterogeneous nuclear ribonucleoprotein A2/B1 (hnRNPA2B1) [75]. These lncRNAs also have clinical relevance, as serum levels of SNHG14 and AFAP-AS1 are considerably elevated in patients exhibiting resistance to therapy, and knockdown of AFAP-AS1 was found to improve patient response to trastuzumab [76].

##### MicroRNAs

Extracellular miRNAs derived from cancer cells can be internalized by cells of the tumor microenvironment wherein they regulate disease-associated processes such as immune response, angiogenesis, metastasis, and drug resistance [77]. Indeed, transfer of cancer cell-derived exosomal microRNAs has been strongly linked to increased drug resistance via regulation of key signaling pathways in recipient cells. Along these lines, several studies have focused on the key role of exosomal miR-21 in mediating drug resistance. In oral squamous cell carcinoma (OSCC), miR-21 is highly expressed in the exosomes derived from cisplatin-resistant cells and is transferred to sensitive cells, thus increasing their cisplatin resistance [78]. In contrast to cisplatin-sensitive cells, exosomes derived from resistant cells cause a decrease in phosphate and tensin homolog (PTEN) and programmed cell death 4 (PDCD4) at both the mRNA and protein level [78]. PTEN and PDCD4 are known tumor suppressor genes. The former acts via the attenuating PTEN/PI3K/AKT signaling pathway and the latter inhibits tumor progression through interacting with elF4A and elF4G [78]. MiR-21 has been shown to regulate proliferation, migration, and resistance to apoptosis through targeting of the PTEN/PI3K/AKT pathway [78]. Injecting exosomes from cisplatin-resistant OSCC cells into a mouse model significantly decreases protein levels of PTEN and PDCD4 in xenografted cancer cells [79]. Interestingly, the PTEN/PI3K/AKT pathway has been found to be targeted by other exosomal microRNAs linked to drug resistance. In hepatocellular carcinoma, miR-32-5p is enriched in exosomes derived from 5-fluorouracil (5-FU)-resistant cells and miR-32-5p overexpression directly inhibits PTEN expression and induces multidrug resistance [80]. Another example, miR-223, is discussed below. In addition to the In addition to the guide strand, the passenger strand miR-21-3p has also been linked to drug resistance. Exosomes secreted from cisplatin-resistant ovarian cancer (OC) cells transmit miR-21-3p to parental cells to confer chemoresistance via downregulating the expression of neuron navigator 3 (NAV3) [81].

There are other miRNAs overexpressed in exosomes secreted from cancer cells and their roles in activating or inhibiting downstream targets and thus developing drug resistance have been shown in various studies. These have been summarized in Table 2.

In addition to those derived from cancer cells, exosomes secreted from cells of the TME can also transfer miRNA cargo to cancer cells and initiate pathways that can lead to chemoresistance. In OC, the expression of miR-21 is significantly higher in cancer-associated adipocytes (CAAs) and CAFs compared to cancer cells [84]. MiR-21 can be transmitted from CAFs and CAAs to cancer cells via exosomes where it directly binds to APAF1 [84]. While binding to its novel target, miR-21 inhibits apoptosis in OC cells and induces chemoresistance [84]. Inhibiting exosomal transfer of miR-21 restores the sensitivity of the cells to chemotherapy and can be used as an alternative treatment for metastatic OC [84]. In addition to miR-21, exosomes of OC CAFs are also enriched with miR-98-5p. Exosomal miR-98-5p can be transferred to cancer cells and induce resistance to cisplatin through direct expression inhibition of cyclin-dependent kinase inhibitor 1A (CDKN1A, p21) [88]. CDKN1A is a member of the Cip/Kip family that plays major roles in cell cycle arrest and its inhibition promotes cell proliferation, apoptosis inhibition, and thus chemoresistance in cisplatin-sensitive OC cells. Moreover, CAF-derived exosomal miR-98-5p inhibits the expression of CDKN1A and promotes resistance to cisplatin in nude mice [88]. Finally, external factors such as hypoxia have been shown to alter EV microRNA cargo in a way that influences drug resistance. Hypoxic cells in OC induce the recruitment of tumor associated macrophages (TAMs) that secrete exosomes high in miR-223. TAM-derived miR-223 can be internalized by epithelial OC cells and promote drug resistance in recipient cells via targeting the PTEN and activating the PI3K/AKT signaling pathway both in vitro and in vivo [87]. Moreover, patients with high hypoxia inducible factor-1a (HIF-1a) expression exhibit significantly higher levels of CD163+ cell infiltration and intertumoral miR-223 [87]. Thus, EVs from cancer cells and cells of the microenvironment have the ability to alter drug resistance.

##### Circular RNAs

Circular RNAs (circRNAs) are single-stranded RNA molecules that form a covalently closed loop and regulate diverse cellular processes [98]. While less well-studied in the context of exosomal drug resistance than non-coding RNAs, circRNAs have also been suggested to mediate therapeutic response in preclinical studies. Exosomal circRNAs are involved in the development of resistance to 5-FU-based treatment in patients with CRC. A recent study identified one hundred and five circRNAs that were significantly upregulated in the exosomes secreted from drug-resistant cell lines as compared to sensitive lines, with potential for developing resistance in recipient cells [99]. In addition, a pair of studies found that exosomal circRNAs from cancer cells could increase drug resistance by altering cellular metabolism [86,96]. In the first, circRNA ciRS-122 was found to act as a sponge to inhibit miR-122, leading to an increase in expression of the M2 isoform of pyruvate kinase that lead to increased drug resistance [86]. Moreover, si-ciRS-122 delivered by exosomes inhibited glycolysis and enhanced drug response in vivo [86]. In addition to ciRS-122, hsa_circRNA_0002130 was found to be overexpressed in exosomes from osimertinib-resistant NSCLC cells [96]. Exosomal hsa_circRNA_0002130 sponged miR-498 and thereby increased the expression levels of GLUT1, HK2, and LDHA. This lead to increased tumor growth and glycolysis and inhibited apoptosis [96]. While it has been demonstrated that circRNAs can impact drug resistance via the alteration of cell metabolism, we anticipate that other mechansisms will be discovered in the coming months and years.

#### 2.5.2. DNA Damage Repair

DNA damage repair (DDR) mechanisms are a major determinant of the response to anticancer therapy. Nucleotide Excision Repair (NER) is an important DDR pathway that drives the removal of abnormal chemical structures in DNA known as DNA lesions, which may be caused by chemotherapeutic drugs [4]. There are two NER sub-pathways: Global Genomic NER (GG-NER), which is responsible for repairing lesions in silent DNA regions, and Transcription-Coupled NER (TC-NER), which repairs DNA damage at transcriptionally active loci [4]. ERCC1 is a gene involved in the NER pathway and its expression correlates with platinum-related DDR [100]. There are also certain proteins such as mismatch repair (MMR) complexes that transduce DNA damage signals caused by platinum-based agents [4]. In renal cell carcinoma, sorafenib-resistant cells exhibit significant levels of exosomal miR-31-5p. MiR-31-5p effectively targets MutL homolog 1 (MLH1)—a protein coding gene involved in protein mismatch repair—and downregulates its expression. These exosomes can be internalized by parental cells and disseminate sorafenib resistance to the whole tumor. Upregulating the expression of MLH1 promotes drug-induced cytotoxicity. In addition, exosomal miR-31-5p plasma levels directly correlate with the patients’ response to sorafenib. Patients with progressive disease exhibit higher plasma expression levels of miR-31-5p during treatment with sorafenib [97].

TP53 is a major effector that initiates cell death and plays a critical role in carcinogenesis when mutated. In many cancers, DNA damage causes cell death through TP53-related genes and mutations in these genes can modulate resistance to therapy [101]. In prostate cancer, exosomes derived from primary prostate fibroblasts promote resistance to chemotherapy. Co-culturing fibroblasts with prostate cancer cells inhibits the expression of P53 via transferring exosomal miR-27a to the recipient cells and downregulation of P53 is linked with increased resistance to cisplatin, doxorubicin, and docetaxel [102].

#### 2.5.3. Drug Activation and Inactivation

Many chemotherapeutic drugs require activation before they become effective, and a lack of activation can decrease or inhibit their effectiveness. By the same token, activated drugs can be inactivated, again making them unable to inhibit tumorigenesis.The mechanism by which drug activation and inactivation occurs is specific to each pharmacologic category of drug and is based on the activity of the enzymes that either convert the drug to its active or nactive form [43]. For example, decreased levels of metabolizing enzyme such as deoxycytidine kinase (DCK)—responsible for converting gemcitabine to its active form—and increased production of reactive oxygen species (ROS) have been reported to be involved in regulating the cytotoxic efficacy of drugs. Exosome-encapsulated miR-155 downregulates the expression of DCK and is also known to promote ROS detoxification by altering the expression of ROS-detoxifying enzymes CAT and SOD2 [93]. These factors induce resistance to gemcitabine in treatment-naïve pancreatic cancer cells [93]. In another study, macrophage-derived exosomes (MDE) were shown to be key regulators of gemcitabine resistance in pancreatic ductal adenocarcinoma (PDAC) and to act via the transmission of miR-365 to cells of the TME [95]. MiR-365 promotes chemoresistance by inducing the enzyme responsible for gemcitabine inactivation, cytidine deaminase, in cancer cells [95]. MiR-365 antagonists in PDAC-bearing mice restore the response to gemcitabine [95]. In conclusion, the exosomal transfer of miR-365 from macrophages to PCAD cells alters the metabolism of gemcitabine and results in chemotherapy resistance.

## 3. Conclusions

Several studies have demonstrated the ability of extracellular vesicles isolated from drug-resistant cancer cells to disseminate anticancer drug resistance via delivering potentially oncogenic molecules to the cells of the tumor microenvironment. It is evident from in vitro and in vivo studies that exosomes induce drug resistance via several mechanisms. More importantly, elevated serum levels of exosomal cargo in patients with poor response to treatment have been detected in various cancer types. Understanding these mechanisms will help us develop novel therapeutic targets as well as non-invasive biomarkers in order to monitor the therapeutic efficacy of anticancer drugs.

## Figures and Tables

**Table 1:** Main features of extracellular vesicles.

	Exosomes	Ectosomes	Apoptotic Bodies
Origin	Endosome	Plasma membrane	Plasma membrane
Size	30–150 nm	50–1000 nm	500–5000 nm
Surface markers	Ceramide, ALIX, CD63, CD9, CD81, Rab5 [39,40]	Integrin-β, CD40 and selectins, CD63, CD9 [41]	Plasma membrane glycoproteins such as alpha-D-mannose and beta-D-galactose, CD63, CD9 [42]

**Table 2:** Summary of anticancer drugs and exosomal cargo involved in drug resistance.

Anticancer Agent	Pharmacologic Category	Cancer Type	Exosomal Content	Cargo Type	Mechanism	Reference
Doxorubicin	Anthracycline	Breast cancer	ABCB1	Protein	Drug efflux	[46]
			TrpC5	Protein	Drug efflux	[47]
			UCH-L1	Protein	Inducing MDR	[48]
			H19	lncRNA	Increased cell viability and colony-forming ability, decreased apoptotic rate	[58]
		ESCC	linc-VLDLR	lncRNA	Upregulating ABCG2	[82]
Paclitaxel	Antimicrotubular	GC	miR-155	miRNA	Suppressing TP53INP and GATA3, inducing EMT	[83]
		OC	miR-21	miRNA	Targeting APAF1	[84]
			miR-1246	miRNA	Altering Cav1/PDGFβ pathway, reducing Cav1 and increasing ABCB1 levels	[85]
Oxaliplatin	Platinum agent	CRC	H19	lncRNA	Inhibiting miR-141, activating Wnt/β-catenin pathway	[60]
			ciRS-122	CircRNA	Sponging miR-122 and inducing PKM2 expression	[86]
Platinum agents	Platinum agent	OC	miR-223	miRNA	Inhibiting PTEN, activating PI3K/AKT pathway	[87]
Cisplatin	Platinum agent	OC	miR-21	miRNA	Downregulating NAV3	[81]
			miR-98-5p	miRNA	Inhibiting CDKN1A	[88]
		Cervical cancer	HNF1A/AS1	lncRNA	Sponging miR-34b, upregulating TUFT1	[62]
		Head and neck cancer	miR-21	miRNA	Inhibiting PTEN and PDCD4	[78]
			miR-196a	miRNA	Targeting CDKN1B and ING5	[89]
			miR-193	miRNA	Targeting FAP2C and activating VEGF and Jak-STAT signaling pathways	[90]
		GC	HOTTIP	lncRNA	Inhibiting miR-218 and increasing HMGA1	[61]
Temozolomide	Alkylating agent	GBM	MET	Protein	Inducing EMT	[54]
			miR-1238	miRNA	Targeting CAV1/EGFR pathway	[91]
Gefitinib	EGFR inhibitor	NSCLC	H19	lncRNA	Increased incorporation into exosomes	[92]
		ESCC	PART1	lncRNA	Sponging miR-129, increasing the expression of Bcl-2	[73]
Trastuzumab	Anti-HER2 monoclonal antibody	HER2+Breast cancer	AFAP1-AS1	lncRNA	Enhancing ERBB2 gene translation	[74]
			AGAP2-AS1	lncRNA	Increased incorporation into exosomes	[75]
			SNHG14	lncRNA	Activating Bcl-2/apoptosis regulator Bax signaling pathway	[76]
Gemcitabine	Antimetabolite	Pancreatic ductal adenocarcinoma	miR-155	miRNA	Inhibiting DCK and ROS detoxification	[93]
			miR-210	miRNA	Activating mTOR pathway	[94]
			miR-365	miRNA	Drug inactivation	[95]
Erlotinib	EGFR inhibitor	NSCLC	H19	lncRNA	Targeting miR-615-3p, upregulating ATG7 expression	[57]
			RP11-838N2.4	lncRNA	Inhibition of apoptosis	[72]
Osimertinib			MSTRG.292666.16	lncRNA	Downregulating miR-21, miR-125b, TGFβ, ARF6. Upregulating c-Kit	[71]
			hsa_circRNA_0002130	CircRNA	Sponging miR-498, inducing GLUT1, HK2 and LDHA expression, increasing glycolysis	[96]
PLX4720	BRAF inhibitor	Melanoma	PDGFRβ	Protein	Activating of PI3K/AKT pathway	[52]
Vemurafenib			ALK^RES^	Protein	Activating MAPK pathway	[53]
Sorafenib	TKI	Renal cell carcinoma	miR-31-5p	miRNA	Downregulating MLH1	[97]
5-FU	Antimetabolite	Hepatocellular carcinoma	miR-32-5p	miRNA	Inhibiting PTEN, promoting EMT, inducing MDR	[80]

ESCC, esophageal squamous cell carcinoma; GC, gastric cancer; CRC, colorectal carcinoma; GBM, glioblastoma multiforme; EGFR, epidermal growth factor receptor; NSCLC, non-small cell lung cancer; BRAF, v-Raf murine sarcoma viral oncogene homolog B; TKI, tyrosine kinase inhibitor.

## Abbreviations

ABC	ATP-binding cassette
AFAP1-AS1	Actin filament associated protein 1 antisense RNA1
ALIX	ALG-2-interacting protein X
ALK	Anaplastic lymphoma kinase
ATG7	Autophagy-related protein 7
AUF1	AU binding factor 1
BCRP	Breast cancer resistance protein
BMPs	Bone morphogenetic proteins
BRAF	v-Raf murine sarcoma viral oncogene homolog B
CAAs	Cancer associated adipocytes
CAFs	Cancer associated fibroblasts
CDKN1A	Cyclin-dependent kinase inhibitor 1A
CeRNA	Competing endogenous RNA
CircRNAs	Circular RNAs
CRC	Colorectal cancer
DCK	Deoxycytidine kinase
DDR	DNA damage repair
DNMTs	DNA methyltransferases
EGFR	Epidermal growth factor receptor
EMT	Epithelial-mesenchymal transition
ERK	extracellular signal-regulated kinase
ESCC	esophageal squamous cell carcinoma
ESCRT	Endosomal sorting complex required for transport
EV	Extracellular vesicle
FOXO1	Forkhead box protein O1
5-FU	5-fluorouracil
GATA3	GATA binding protein 3
GBM	Glioblastoma multiforme
GC	Gastric Cancer
GG-NER	Global Genomic NER
HMGA1	High mobility group AT-hook 1
hnRNPA1	Heterogeneous nuclear ribonucleoprotein A1
hnRNPA2B1	Heterogeneous nuclear ribonucleoprotein A2B1
HOTTIP	HOXA transcript at the distal tip
HSP	Heat shock protein
ILV	Intraluminal vesicles
ING5	Inhibitor of Growth Family Member 5
lncRNA	Long non-coding RNAs
MAPK/ERK	Mitogen-activated protein kinase/extracellular receptor kinase
MDE	Macrophage-derived exosomes
MDR	Multidrug resistance
MET	Mesenchymal Epithelial Transition
MGMT	O_6_-methylguanyl DNA methyltransferase
miRNAs	MicroRNAs
MLH1	MutL homolog 1
MMR	Mismatch repair
MRP1	MDR-associated protein1
mTOR	Mammalian target of rapamycin
MV	Microvesicles
MVB	Multivesicular bodies
NAV3	Neuron navigator 3
NER	Nucleotide excision repair
NSCLC	Non-small cell lung cancer
nSMase	neutral sphingomyelinases
OC	Ovarian cancer
OSCC	Oral squamous cell carcinoma
PART1	Prostate androgen-regulated transcript 1
PDAC	Pancreatic ductal adenocarcinoma
PDCD4	Programmed cell death 4
PDGFRβ	Platelet-derived growth factor receptor beta
PI3K	Phosphatidylinositol 3-kinase
PKM2	M2 isoform of pyruvate kinase
PTEN	Phosphate and tensin homolog
RBP	RNA binding proteins
RNP	Ribonucleoprotein
ROS	Reactive oxygen species
siRNA	Small interfering RNA
SNHG14	Small nucleolar RNA host gene 14
STAT1	Signal transducer and activator of transcription 1
TAM	Tumor associated macrophages
TC-NER	Transcription-coupled nucleotide excision repair
TKI	Tyrosine kinase inhibitor
TME	Tumor microenvironment
TP53INP1	p53-inducible nuclear protein 1
TrpC5	Transient receptor potential channel 5
TSG101	Tumor-susceptibility gene-101
TUFT1	Tuftelin1
3′-UTR	3′-untranslated region
UCH-L1	Ubiquitin carboxy-terminal hydrolase L1
UGT1A1	UDP glucuronosyltransferase 1

## References

[1] Siegel R.L., Miller K.D., Sauer A.G., Fedewa S.A., Butterly L.F., Anderson J.C., Cercek A., Smith R.A., Jemal A. (2020). Colorectal cancer statistics, 2020. CA Cancer J. Clin..

[2] Joo W.D., Visintin I., Mor G. (2013). Targeted cancer therapy–are the days of systemic chemotherapy numbered?. Maturitas.

[3] Sharma A. (2017). Chemoresistance in cancer cells: Exosomes as potential regulators of therapeutic tumor heterogeneity. Nanomedicine.

[4] Nikolaou M., Pavlopoulou A., Georgakilas A.G., Kyrodimos E. (2018). The challenge of drug resistance in cancer treatment: A current overview. Clin. Exp. Metastasis.

[5] Hu X., Zhang Z. (2016). Understanding the genetic mechanisms of cancer drug resistance using genomic approaches. Trends Genet..

[6] Jabalee J., Towle R., Garnis C. (2018). The role of extracellular vesicles in cancer: Cargo, function, and therapeutic implications. Cells.

[7] Peinado H., Zhang H., Matei I.R., Costa-Silva B., Hoshino A., Rodrigues G., Psaila B., Kaplan R.N., Bromberg J.F., Kang Y. (2017). Pre-metastatic niches: Organ-specific homes for metastases. Nat. Rev. Cancer.

[8] Maacha S., Bhat A.A., Jimenez L., Raza A., Haris M., Uddin S., Grivel J.-C. (2019). Extracellular vesicles-mediated intercellular communication: Roles in the tumor microenvironment and anti-cancer drug resistance. Mol.Cancer.

[9] Wortzel I., Dror S., Kenific C.M., Lyden D. (2019). Exosome-mediated metastasis: Communication from a distance. Dev. Cell.

[10] Mathieu M., Martin-Jaular L., Lavieu G., Thery C. (2019). Specificities of secretion and uptake of exosomes and other extracellular vesicles for cell-to-cell communication. Nat. Cell Biol..

[11] Hsu C., Morohashi Y., Yoshimura S.-I., Manrique-Hoyos N., Jung S., Lauterbach M.A., Bakhti M., Grønborg M., Möbius W., Rhee J. (2010). Regulation of exosome secretion by Rab35 and its GTPase-activating proteins TBC1D10A–C. J. Cell Biol..

[12] Pfeffer S.R. (2010). Two Rabs for exosome release. Nat. Cell Biol..

[13] Schuh A.L., Audhya A. (2014). The ESCRT machinery: From the plasma membrane to endosomes and back again. Crit. Rev. Biochem. Mol. Biol..

[14] Lopatina T., Gai C., Deregibus M.C., Kholia S., Camussi G. (2016). Cross talk between cancer and mesenchymal stem cells through extracellular vesicles carrying nucleic acids. Front. Oncol..

[15] Liao J., Liu R., Shi Y.-J., Yin L.-H., Pu Y.-P. (2016). Exosome-shuttling microRNA-21 promotes cell migration and invasion-targeting PDCD4 in esophageal cancer. Int. J. Oncol..

[16] Webber J., Clayton A. (2013). How pure are your vesicles?. J. Extracell. Vesicles.

[17] Valadi H., Ekström K., Bossios A., Sjöstrand M., Lee J.J., Lötvall J.O. (2007). Exosome-mediated transfer of mRNAs and microRNAs is a novel mechanism of genetic exchange between cells. Nat. Cell Biol..

[18] Simons M., Raposo G. (2009). Exosomes-vesicular carriers for intercellular communication. Curr. Opin. Cell Biol..

[19] McKenzie A.J., Hoshino D., Hong N.H., Cha D.J., Franklin J.L., Coffey R.J., Patton J.G., Weaver A.M. (2016). KRAS-MEK signaling controls Ago2 sorting into exosomes. Cell Rep..

[20] Takasugi M., Okada R., Takahashi A., Chen D.V., Watanabe S., Hara E. (2017). Small extracellular vesicles secreted from senescent cells promote cancer cell proliferation through EphA2. Nat. Commun..

[21] Itoh S., Mizuno K., Aikawa M., Aikawa E. (2018). Dimerization of sortilin regulates its trafficking to extracellular vesicles. J. Biol. Chem..

[22] Perez-Hernandez D., Gutiérrez-Vázquez C., Jorge I., López-Martín S., Ursa A., Sánchez-Madrid F., Vázquez J., Yáñez-Mó M. (2013). The intracellular interactome of tetraspanin-enriched microdomains reveals their function as sorting machineries toward exosomes. J. Biol. Chem..

[23] Wubbolts R., Leckie R.S., Veenhuizen P.T., Schwarzmann G., Möbius W., Hoernschemeyer J., Slot J.W., Geuze H.J., Stoorvogel W. (2003). Proteomic and biochemical analyses of human B cell-derived exosomes Potential implications for their function and multivesicular body formation. J. Biol. Chem..

[24] Wilusz J.E., Sunwoo H., Spector D.L. (2009). Long noncoding RNAs: Functional surprises from the RNA world. Genes Dev..

[25] Kai K., Dittmar R.L., Sen S. (2018). Secretory microRNAs as biomarkers of cancer. Seminars in Cell & Developmental Biology.

[26] Wang M., Zhou L., Yu F., Zhang Y., Li P., Wang K. (2019). The functional roles of exosomal long non-coding RNAs in cancer. Cel. Mol. Life Sci..

[27] Villarroya-Beltri C., Gutiérrez-Vázquez C., Sánchez-Cabo F., Pérez-Hernández D., Vázquez J., Martin-Cofreces N., Martinez-Herrera D.J., Pascual-Montano A., Mittelbrunn M., Sánchez-Madrid F. (2013). Sumoylated hnRNPA2B1 controls the sorting of miRNAs into exosomes through binding to specific motifs. Nat. Commun..

[28] Mukherjee K., Ghoshal B., Ghosh S., Chakrabarty Y., Shwetha S., Das S., Bhattacharyya S.N. (2016). Reversible HuR-micro RNA binding controls extracellular export of miR-122 and augments stress response. EMBO Rep..

[29] Statello L., Maugeri M., Garre E., Nawaz M., Wahlgren J., Papadimitriou A., Lundqvist C., Lindfors L., Collén A., Sunnerhagen P. (2018). Identification of RNA-binding proteins in exosomes capable of interacting with different types of RNA: RBP-facilitated transport of RNAs into exosomes. PLoS ONE.

[30] Hagiwara K., Katsuda T., Gailhouste L., Kosaka N., Ochiya T. (2015). Commitment of Annexin A2 in recruitment of microRNAs into extracellular vesicles. FEBS Lett..

[31] Koppers-Lalic D., Hackenberg M., Bijnsdorp I.V., van Eijndhoven M.A., Sadek P., Sie D., Zini N., Middeldorp J.M., Ylstra B., de Menezes R.X. (2014). Nontemplated nucleotide additions distinguish the small RNA composition in cells from exosomes. Cell Rep..

[32] Song J., Song J., Mo B., Chen X. (2015). Uridylation and adenylation of RNAs. Sci. China Life Sci..

[33] Squadrito M., Baer C., Burdet F., Maderna C., Gilfillan G., Lyle R. (2014). Endogenous RNAs modulate microRNA sorting to exosomes and transfer to acceptor cells. Cell Rep..

[34] Kubota S., Chiba M., Watanabe M., Sakamoto M., Watanabe N. (2015). Secretion of small/microRNAs including miR-638 into extracellular spaces by sphingomyelin phosphodiesterase 3. Oncol. Rep..

[35] Kosaka N., Iguchi H., Hagiwara K., Yoshioka Y., Takeshita F., Ochiya T. (2013). Neutral sphingomyelinase 2 (nSMase2)-dependent exosomal transfer of angiogenic microRNAs regulate cancer cell metastasis. J. Biol. Chem..

[36] Janas T., Janas M.M., Sapoń K., Janas T. (2015). Mechanisms of RNA loading into exosomes. FEBS Lett..

[37] Zhang G., Zhang Y., Cheng S., Wu Z., Liu F., Zhang J. (2017). CD133 positive U87 glioblastoma cells-derived exosomal microRNAs in hypoxia-versus normoxia-microenviroment. J. Neuro Oncol..

[38] Yogev O., Henderson S., Hayes M.J., Marelli S.S., Ofir-Birin Y., Regev-Rudzki N., Herrero J., Enver T. (2017). Herpesviruses shape tumour microenvironment through exosomal transfer of viral microRNAs. PLoS Pathog..

[39] Kastelowitz N., Yin H. (2014). Exosomes and microvesicles: Identification and targeting by particle size and lipid chemical probes. Chembiochem Eur. J. Chem. Biol..

[40] Van Niel G., d’Angelo G., Raposo G. (2018). Shedding light on the cell biology of extracellular vesicles. Nat. Rev. Mol. Cell Biol..

[41] Anthony D.F., Shiels P.G. (2013). Exploiting paracrine mechanisms of tissue regeneration to repair damaged organs. Transplant. Res..

[42] Bilyy R., Stoika R. (2007). Search for novel cell surface markers of apoptotic cells. Autoimmunity.

[43] Holohan C., van Schaeybroeck S., Longley D.B., Johnston P.G. (2013). Cancer drug resistance: An evolving paradigm. Nat. Rev. Cancer.

[44] Sui H., Fan Z., Li Q. (2012). Signal transduction pathways and transcriptional mechanisms of ABCB1/Pgp-mediated multiple drug resistance in human cancer cells. J. Int. Med. Res..

[45] Sedláková I., Laco J., Caltová K., Cervinka M., Tošner J., Rezác A., Špacek J. (2015). Clinical significance of the resistance proteins LRP, Pgp, MRP1, MRP3, and MRP5 in epithelial ovarian cancer. Int. J. Gynecol. Cancer.

[46] Lv M.-M., Zhu X.-Y., Chen W.-X., Zhong S.-L., Hu Q., Ma T.-F., Zhang J., Chen L., Tang J.H., Zhao J.H. (2014). Exosomes mediate drug resistance transfer in MCF-7 breast cancer cells and a probable mechanism is delivery of P-glycoprotein. Tumor Biol..

[47] Ma X., Chen Z., Hua D., He D., Wang L., Zhang P., Wang J., Cai Y., Gao C., Zhang X. (2014). Essential role for TrpC5-containing extracellular vesicles in breast cancer with chemotherapeutic resistance. Proc. Natl. Acad. Sci. USA.

[48] Ning K., Wang T., Sun X., Zhang P., Chen Y., Jin J., Hua D. (2017). UCH-L1-containing exosomes mediate chemotherapeutic resistance transfer in breast cancer. J. Surg. Oncol..

[49] Hu-Lieskovan S., Mok S., Moreno B.H., Tsoi J., Robert L., Goedert L., Pinheiro E.M., Koya R.C., Graeber T.G., Comin-Anduix B. (2015). Improved antitumor activity of immunotherapy with BRAF and MEK inhibitors in BRAFV600E melanoma. Sci. Transl. Med..

[50] Chapman P.B., Hauschild A., Robert C., Haanen J.B., Ascierto P., Larkin J., Dummer R., Garbe C., Testori A., Maio M. (2011). Improved survival with vemurafenib in melanoma with BRAF V600E mutation. N. Engl. J. Med..

[51] Ascierto P.A., Kirkwood J.M., Grob J.-J., Simeone E., Grimaldi A.M., Maio M., Palmieri G., Testori A., Marincola F.M., Mozzillo N. (2012). The role of BRAF V600 mutation in melanoma. J. Transl. Med..

[52] Vella L.J., Behren A., Coleman B., Greening D.W., Hill A.F., Cebon J. (2017). Intercellular resistance to BRAF inhibition can be mediated by extracellular vesicle–associated PDGFRβ. Neoplasia.

[53] Cesi G., Philippidou D., Kozar I., Kim Y.J., Bernardin F., van Niel G., Wienecke-Baldacchino A., Felten P., Letellier E., Dengler S. (2018). A new ALK isoform transported by extracellular vesicles confers drug resistance to melanoma cells. Mol. Cancer.

[54] Zeng A., Yan W., Liu Y., Wang Z., Hu Q., Nie E., Zhou X., Li R., Wang X.-F., Jiang T. (2017). Tumour exosomes from cells harbouring PTPRZ1–MET fusion contribute to a malignant phenotype and temozolomide chemoresistance in glioblastoma. Oncogene.

[55] Meng F.-D., Wei J.-C., Qu K., Wang Z.-X., Wu Q.-F., Tai M.-H., Liu H.C., Zhang R.Y., Liu C. (2015). FoxM1 overexpression promotes epithelial-mesenchymal transition and metastasis of hepatocellular carcinoma. World J. Gastroenterol. WJG.

[56] Calin G.A., Croce C.M. (2006). MicroRNA signatures in human cancers. Nat. Rev. Cancer.

[57] Pan R., Zhou H. (2020). Exosomal Transfer of lncRNA H19 Promotes Erlotinib Resistance in Non-Small Cell Lung Cancer via miR-615-3p/ATG7 Axis. Cancer Manag. Res..

[58] Wang X., Pei X., Guo G., Qian X., Dou D., Zhang Z., Xu X., Duan X. (2020). Exosome-mediated transfer of long noncoding RNA H19 induces doxorubicin resistance in breast cancer. J. Cell. Physiol..

[59] Yun C.W., Lee S.H. (2018). The roles of autophagy in cancer. Int. J. Mol. Sci..

[60] Ren J., Ding L., Zhang D., Shi G., Xu Q., Shen S., Wang Y., Wang T., Hou Y. (2018). Carcinoma-associated fibroblasts promote the stemness and chemoresistance of colorectal cancer by transferring exosomal lncRNA H19. Theranostics.

[61] Wang J., Lv B., Su Y., Wang X., Bu J., Yao L. (2019). Exosome-mediated transfer of lncRNA HOTTIP promotes cisplatin resistance in gastric cancer cells by regulating HMGA1/miR-218 axis. OncoTargets Ther..

[62] Luo X., Wei J., Yang F.-L., Pang X.-X., Shi F., Wei Y.-X., Liao B.Y., Wang J.L. (2019). Exosomal lncRNA HNF1A-AS1 affects cisplatin resistance in cervical cancer cells through regulating microRNA-34b/TUFT1 axis. Cancer Cell Int..

[63] Tie J., Pan Y., Zhao L., Wu K., Liu J., Sun S., Guo X., Wang B., Gang Y., Zhang Y. (2010). MiR-218 inhibits invasion and metastasis of gastric cancer by targeting the Robo1 receptor. PLoS Genet..

[64] Wang G., Fu Y., Liu G., Ye Y., Zhang X. (2016). miR-218 inhibits proliferation, migration, and EMT of gastric cancer cells by targeting WASF3. Oncol. Res..

[65] Deng M., Zeng C., Lu X., He X., Zhang R., Qiu Q., Zheng G., Jia X., Liu H., He Z. (2017). miR-218 suppresses gastric cancer cell cycle progression through the CDK6/Cyclin D1/E2F1 axis in a feedback loop. Cancer Lett..

[66] Zhang X.-L., Shi H.-J., Wang J.-P., Tang H.-S., Cui S.-Z. (2015). MiR-218 inhibits multidrug resistance (MDR) of gastric cancer cells by targeting Hedgehog/smoothened. Int. J. Clin. Exp. Pathol..

[67] Lodygin D., Tarasov V., Epanchintsev A., Berking C., Knyazeva T., Körner H., Knyazev P., Diebold J., Hermeking H. (2008). Inactivation of miR-34a by aberrant CpG methylation in multiple types of cancer. Cell Cycle.

[68] Bommer G.T., Gerin I., Feng Y., Kaczorowski A.J., Kuick R., Love R.E., Zhai Y., Giordano T.J., Qin Z.S., Moore B.B. (2007). p53-mediated activation of miRNA34 candidate tumor-suppressor genes. Curr. Biol..

[69] Xiong S., Hu M., Li C., Zhou X., Chen H. (2019). Role of miR-34 in gastric cancer: From bench to bedside. Oncol. Rep..

[70] Wu G., Zhou H., Li D., Zhi Y., Liu Y., Li J., Wang F. (2020). LncRNA DANCR upregulation induced by TUFT1 promotes malignant progression in triple negative breast cancer via miR-874-3p-SOX2 axis. Exp. Cell Res..

[71] Deng Q., Fang Q., Xie B., Sun H., Bao Y., Zhou S. (2020). Exosomal long non-coding RNA MSTRG. 292666.16 is associated with osimertinib (AZD9291) resistance in non-small cell lung cancer. Aging.

[72] Zhang W., Cai X., Yu J., Lu X., Qian Q., Qian W. (2018). Exosome-mediated transfer of lncRNA RP11-838N2. 4 promotes erlotinib resistance in non-small cell lung cancer. Int. J. Oncol..

[73] Kang M., Ren M., Li Y., Fu Y., Deng M., Li C. (2018). Exosome-mediated transfer of lncRNA PART1 induces gefitinib resistance in esophageal squamous cell carcinoma via functioning as a competing endogenous RNA. J. Exp. Clin. Cancer Res..

[74] Han M., Gu Y., Lu P., Li J., Cao H., Li X., Qian X., Yu C., Yang Y., Yang X. (2020). Exosome-mediated lncRNA AFAP1-AS1 promotes trastuzumab resistance through binding with AUF1 and activating ERBB2 translation. Mol. Cancer.

[75] Zheng Z., Chen M., Xing P., Yan X., Xie B. (2019). Increased expression of exosomal AGAP2-AS1 (AGAP2 Antisense RNA 1) in breast cancer cells inhibits trastuzumab-induced cell cytotoxicity. Med. Sci. Monit. Int. Med. J. Exp. Clin. Res..

[76] Dong H., Wang W., Chen R., Zhang Y., Zou K., Ye M., He X., Zhang F., Han J. (2018). Exosome-mediated transfer of lncRNA-SNHG14 promotes trastuzumab chemoresistance in breast cancer. Int. J. Oncol..

[77] Zhang C., Ji Q., Yang Y., Li Q., Wang Z. (2018). Exosome: Function and role in cancer metastasis and drug resistance. Technol. Cancer Res. Treat..

[78] Wu Y.-R., Qi H.-J., Deng D.-F., Luo Y.-Y., Yang S.-L. (2016). MicroRNA-21 promotes cell proliferation, migration, and resistance to apoptosis through PTEN/PI3K/AKT signaling pathway in esophageal cancer. Tumor Biol..

[79] Liu T., Chen G., Sun D., Lei M., Li Y., Zhou C., Li X., Xue W., Wang H., Liu C. (2017). Exosomes containing miR-21 transfer the characteristic of cisplatin resistance by targeting PTEN and PDCD4 in oral squamous cell carcinoma. Acta Biochim. Biophys. Sin..

[80] Fu X., Liu M., Qu S., Ma J., Zhang Y., Shi T., Wen H., Yang Y., Wang S., Wang J. (2018). Exosomal microRNA-32-5p induces multidrug resistance in hepatocellular carcinoma via the PI3K/Akt pathway. J. Exp. Clin. Cancer Res..

[81] Pink R.C., Samuel P., Massa D., Caley D.P., Brooks S.A., Carter D.R.F. (2015). The passenger strand, miR-21-3p, plays a role in mediating cisplatin resistance in ovarian cancer cells. Gynecol. Oncol..

[82] Chen Y., Liu L., Li J., Du Y., Wang J., Liu J. (2019). Effects of long noncoding RNA (linc-VLDLR) existing in extracellular vesicles on the occurrence and multidrug resistance of esophageal cancer cells. Pathol. Res. Pract..

[83] Wang M., Qiu R., Yu S., Xu X., Li G., Gu R., Tan C., Zhu W., Shen B. (2019). Paclitaxel-resistant gastric cancer MGC-803 cells promote epithelial-to-mesenchymal transition and chemoresistance in paclitaxel-sensitive cells via exosomal delivery of miR-155-5p. Int. J. Oncol..

[84] Yeung C.L.A., Co N.-N., Tsuruga T., Yeung T.-L., Kwan S.-Y., Leung C.S., Li Y., Lu E.S., Kwan K., Wong K.-K. (2016). Exosomal transfer of stroma-derived miR21 confers paclitaxel resistance in ovarian cancer cells through targeting APAF1. Nat. Commun..

[85] Kanlikilicer P., Bayraktar R., Denizli M., Rashed M.H., Ivan C., Aslan B., Mitra R., Karagoz K., Bayraktar E., Zhang X. (2018). Exosomal miRNA confers chemo resistance via targeting Cav1/p-gp/M2-type macrophage axis in ovarian cancer. EBioMedicine.

[86] Wang X., Zhang H., Yang H., Bai M., Ning T., Deng T., Liu R., Fan Q., Zhu K., Li J. (2020). Exosome-delivered circRNA promotes glycolysis to induce chemoresistance through the miR-122-PKM2 axis in colorectal cancer. Mol. Oncol..

[87] Zhu X., Shen H., Yin X., Yang M., Wei H., Chen Q., Feng F., Liu Y., Xu W., Li Y. (2019). Macrophages derived exosomes deliver miR-223 to epithelial ovarian cancer cells to elicit a chemoresistant phenotype. J. Exp. Clin. Cancer Res..

[88] Guo H., Ha C., Dong H., Yang Z., Ma Y., Ding Y. (2019). Cancer-associated fibroblast-derived exosomal microRNA-98-5p promotes cisplatin resistance in ovarian cancer by targeting CDKN1A. Cancer Cell Int..

[89] Qin X., Guo H., Wang X., Zhu X., Yan M., Wang X., Xu Q., Shi J., Lu E., Chen W. (2019). Exosomal miR-196a derived from cancer-associated fibroblasts confers cisplatin resistance in head and neck cancer through targeting CDKN1B and ING5. Genome Biol..

[90] Shi S., Huang X., Ma X., Zhu X., Zhang Q. (2020). Research of the mechanism on miRNA193 in exosomes promotes cisplatin resistance in esophageal cancer cells. PLoS ONE.

[91] Yin J., Zeng A., Zhang Z., Shi Z., Yan W., You Y. (2019). Exosomal transfer of miR-1238 contributes to temozolomide-resistance in glioblastoma. EBioMedicine.

[92] Lei Y., Guo W., Chen B., Chen L., Gong J., Li W. (2018). Tumor-released lncRNA H19 promotes gefitinib resistance via packaging into exosomes in non-small cell lung cancer. Oncol. Rep..

[93] Patel G.K., Khan M.A., Bhardwaj A., Srivastava S.K., Zubair H., Patton M.C., Singh S., Khushman M., Singh A.P. (2017). Exosomes confer chemoresistance to pancreatic cancer cells by promoting ROS detoxification and miR-155-mediated suppression of key gemcitabine-metabolising enzyme, DCK. Br. J. Cancer.

[94] Yang Z., Zhao N., Cui J., Wu H., Xiong J., Peng T. (2020). Exosomes derived from cancer stem cells of gemcitabine-resistant pancreatic cancer cells enhance drug resistance by delivering miR-210. Cell. Oncol..

[95] Binenbaum Y., Fridman E., Yaari Z., Milman N., Schroeder A., David G.B., Shlomi T., Gil Z. (2018). Transfer of miRNA in macrophage-derived exosomes induces drug resistance in pancreatic adenocarcinoma. Cancer Res..

[96] Ma J., Qi G., Li L. (2020). A Novel Serum Exosomes-Based Biomarker hsa_circ_0002130 Facilitates Osimertinib-Resistance in Non-Small Cell Lung Cancer by Sponging miR-498. OncoTargets Ther..

[97] He J., He J., Min L., He Y., Guan H., Wang J., Peng X. (2020). Extracellular vesicles transmitted miR-31-5p promotes sorafenib resistance by targeting MLH1 in renal cell carcinoma. Int. J. Cancer.

[98] Yu C.-Y., Kuo H.-C. (2019). The emerging roles and functions of circular RNAs and their generation. J. Biomed. Sci..

[99] Hon K.W., Ab-Mutalib N.S., Abdullah N.M.A., Jamal R., Abu N. (2019). Extracellular Vesicle-derived circular RNAs confers chemoresistance in Colorectal cancer. Sci. Rep..

[100] Stubbert L.J., Smith J.M., McKay B.C. (2010). Decreased transcription-coupled nucleotide excision repair capacity is associated with increased p53-and MLH1-independent apoptosis in response to cisplatin. BMC Cancer.

[101] Siddik Z.H. (2003). Cisplatin: Mode of cytotoxic action and molecular basis of resistance. Oncogene.

[102] Cao Z., Xu L., Zhao S. (2019). Exosome-derived miR-27a produced by PSC-27 cells contributes to prostate cancer chemoresistance through p53. Biochem. Biophys. Res. Commun..

